# Non-Volatile Ferroelectric Switching of Ferromagnetic Resonance in NiFe/PLZT Multiferroic Thin Film Heterostructures

**DOI:** 10.1038/srep32408

**Published:** 2016-09-01

**Authors:** Zhongqiang Hu, Xinjun Wang, Tianxiang Nan, Ziyao Zhou, Beihai Ma, Xiaoqin Chen, John G. Jones, Brandon M. Howe, Gail J. Brown, Yuan Gao, Hwaider Lin, Zhiguang Wang, Rongdi Guo, Shuiyuan Chen, Xiaoling Shi, Wei Shi, Hongzhi Sun, David Budil, Ming Liu, Nian X. Sun

**Affiliations:** 1 Department of Electrical and Computer Engineering, Northeastern University, Boston, Massachusetts 02115, USA; 2 Materials and Manufacturing Directorate, Air Force Research Laboratory, Wright-Patterson Air Force Base, Ohio 45433, USA; 3 Electronic Materials Research Laboratory, Key Laboratory of the Ministry of Education & International Center for Dielectric Research, Xi’an Jiaotong University, Xi’an 710049, China; 4 Energy Systems Division, Argonne National Laboratory, Argonne, Illinois 60439, USA; 5 Department of Chemistry, Northeastern University, Boston, Massachusetts 02115, USA

## Abstract

Magnetoelectric effect, arising from the interfacial coupling between magnetic and electrical order parameters, has recently emerged as a robust means to electrically manipulate the magnetic properties in multiferroic heterostructures. Challenge remains as finding an energy efficient way to modify the distinct magnetic states in a reliable, reversible, and non-volatile manner. Here we report ferroelectric switching of ferromagnetic resonance in multiferroic bilayers consisting of ultrathin ferromagnetic NiFe and ferroelectric Pb_0.92_La_0.08_Zr_0.52_Ti_0.48_O_3_ (PLZT) films, where the magnetic anisotropy of NiFe can be electrically modified by low voltages. Ferromagnetic resonance measurements confirm that the interfacial charge-mediated magnetoelectric effect is dominant in NiFe/PLZT heterostructures. Non-volatile modification of ferromagnetic resonance field is demonstrated by applying voltage pulses. The ferroelectric switching of magnetic anisotropy exhibits extensive applications in energy-efficient electronic devices such as magnetoelectric random access memories, magnetic field sensors, and tunable radio frequency (RF)/microwave devices.

Recent development of spintronic memory, logic, and signal processing devices requires direct manipulation of magnetism by electric field, which is more power efficient and scalable than by magnetic field. Voltage-induced magnetism change arising from coupling between the magnetic and electrical order parameters (i.e., magnetoelectric (ME) coupling) in magnetic/dielectric composites has recently emerged as a robust means of controlling magnetic properties in the adjacent magnetic layer^1^,^2^,^3^,^4^,^5^,^6^,^7^,^8^,^9^. In particular, the magnetoelectric coupling has been demonstrated to modify the magnetic properties as varied as magnetic ordering^10^, magnetic anisotropy^11^,^12^,^13^,^14^,^15^,^16^,^17^,^18^,^19^,^20^,^21^, spin polarization^22^, Curie temperature^23^, and ferromagnetic resonance^24^,^25^. Several mechanisms including strain-, charge-, and exchange bias-mediated effects have been identified in different magnetoelectric heterostructures^26^,^27^,^28^,^29^,^30^,^31^,^32^.

Strong ME coupling has been achieved in magnetostrictive/piezoelectric composites using high-quality piezoelectric single crystals such as BaTiO_3_^33^,^34^, lead magnesium niobate-lead titanate (PMN-PT)^35^,^36^, lead zinc niobate-lead titanate (PZN-PT)^19^, and lead indium niobate-lead magnesium niobate-lead titanate (PIN-PMN-PT)^37^, where the voltage applied to the piezoelectric substrates exerts a mechanical deformation on the magnetostrictive materials, and thus produces a strain-mediated ME effect^35^,^36^,^37^,^38^,^39^. Generally, the piezo-crystal used in these heterostructures has a thickness of ~0.5 mm, which requires a high operating voltage of ~400 V to generate a strong mechanical coupling. Although piezoelectric thin films can be grown by carefully optimizing the deposition process, the weak piezoelectric strength due to substrate clamping effect has hindered the facile fabrication of reliable and compact devices. Attention has therefore been turned to magnetic/high-*k* dielectric bilayers such as Fe/MgO^11^,^40^, CoFe/MgO^41^,^42^, Co/GdO_x_^20^, and NiFe/SrTiO_3_^43^, etc., in which the magnetic anisotropy depends sensitively on voltage-driven charge accumulation or ionic migration at the interface. In general, the ME coupling based on high-*k* dielectric is inherently volatile, despite its outstanding performance and compatibility with Si substrates. Of particular recent scientific and technological interests are ferromagnetic/ferroelectric multiferroic bilayers, such as LSMO/PZT^28^, Co/P(VDF-TrFE)^12^, and CoFe/BST^44^, where the functionality is similar to recently proposed magnetic/high-*k* dielectric stacks but the associated volatile challenges have been addressed by replacing the dielectric layer with a well-established charge-screening ferroelectric thin film. In these multiferroic bilayers, the remanent polarization in ferroelectric layer offers a convenient source of switchable charges, and satisfies the need for low power-consumption, non-volatile behaviour in electrically controlled magnetic devices. Nevertheless, the charge-mediated ME effect in multiferroic bilayers has suffered from small coupling coefficient caused by low remanent polarization, and the understanding of ferroelectric switching of ferromagnetic resonance has been limited^9^.

In this work, we report the ferroelectric switching of ferromagnetic resonance (FMR) in multiferroic bilayers consisting of ultrathin ferromagnetic Ni_80_Fe_20_ (NiFe) and ferroelectric Pb_0.92_La_0.08_Zr_0.52_Ti_0.48_O_3_ (PLZT) films, where a large magnetic anisotropy change of 1.7 μJ/m^2^ is demonstrated under the application of ±10 V voltage pulses at room temperature. Moreover, angular-dependent ferromagnetic resonance measurement is utilized to investigate the interfacial charge-mediated ME effect, providing a mechanism to distinguish the origin of complex and subtle ME coupling in multiferroic bilayers.

## Results

**Figure 1:**
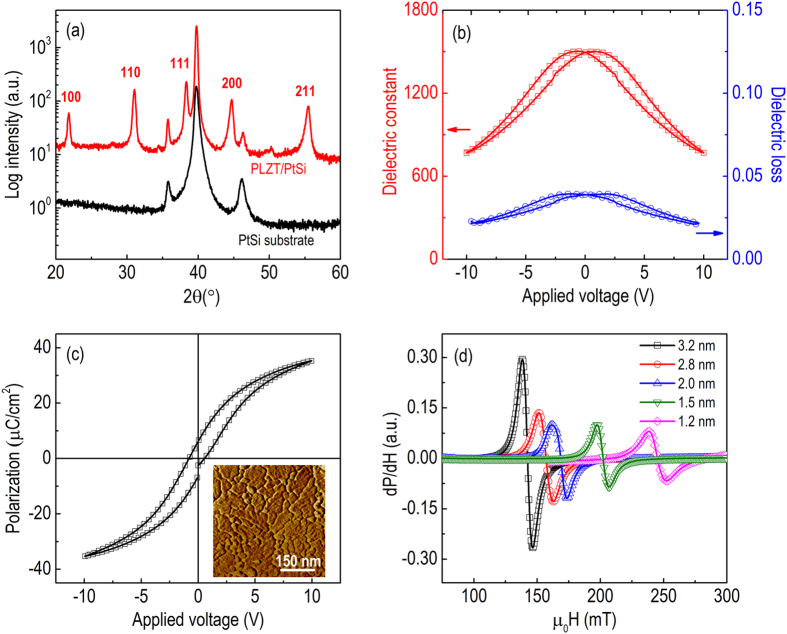
Structural, electrical, and ferromagnetic properties of NiFe/PLZT heterostructures. (**a**) X-ray diffraction patterns of PLZT thin films grown on PtSi Substrates showing a preferred (111) orientation. (**b**) Voltage-dependent dielectric constant and dielectric loss of PLZT. (**c**) Polarization-voltage hysteresis loop and surface morphology of PLZT. (**d**) Ferromagnetic resonance spectra of NiFe/PLZT bilayers with various NiFe thicknesses t_N_.

PLZT is selected as the ferroelectric material because of its relatively low coercive field and high remanent polarization, which would reduce the power consumption and enhance the ME coupling. Structural, electrical, and morphological properties of PLZT are summarized in Fig. 1(a–c). X-ray diffraction pattern of the 350 nm thick PLZT films is indexed by a pseudocubic structure and compared with that of the PtSi substrates, as shown in Fig. 1(a). Well-crystallized perovskite structure is confirmed in the PLZT samples without any traceable secondary phase. The high intensity of (111) peak indicates a preferred (111) orientation in PLZT thin films, consistent with previous report^45^. The dielectric constant and loss of PLZT are shown in Fig. 1(b) as a function of applied voltage, which display typical butterfly shaped hysteresis. At zero voltage bias, a high dielectric constant of 1500 and a low dielectric loss of 0.04 are observed for PLZT films on PtSi substrates. Under an applied voltage of 10 V, the dielectric constant and loss decreases to 760 and 0.02, respectively. Figure 1(c) shows the polarization-voltage (P-V) hysteresis loop at a maximum applied voltage of 10 V and a frequency of 100 Hz. The saturation polarization (*P*_*s*_) and remanent polarization (*P*_*r*_) are measured as 35 and 7 μC/cm^2^, respectively. The inset of Fig. 1(c) presents the surface morphology of PLZT examined by atomic force microscopy (AFM). A well-defined smooth surface is observed with grain size in the range of 20–100 nm. The root-mean-square roughness (R_ms_) of the PLZT surface obtained by AFM is ~0.9 nm, which is further reduced by plasma etching before NiFe deposition. These results demonstrate superior electrical performance of PLZT thin film, making it a suitable ferroelectric layer for improving the ME coupling in multiferroic heterostructures.

Magnetically soft alloy of Ni_80_Fe_20_ (NiFe) is selected as the ferromagnetic material because of its narrow resonance linewidth that enables precise detection of small voltage-induced resonance modification^38^. NiFe dots with a diameter of 500 μm and nominal thicknesses t_N_ = 1.2, 1.5, 2.0, 2.8, and 3.2 nm were prepared on the PLZT films through a shadow mask by magnetron sputtering. Figure 1(d) shows the FMR spectra of the NiFe/PLZT bilayer with various t_N_, measured with the applied magnetic field in the film plane. The decrease in t_N_ leads to an increase in resonance field μ_0_H_FMR_, due to the change in magnetic anisotropy. When t_N_ is reduced to below 1.2 nm, the resonance signal deteriorates with a very broad linewidth (W_pp_ > 50 mT), due to the increased damping induced by interfacial defects and surface roughness. Therefore, we could not detect any systematic resonance field change in NiFe with t_N_ < 1.2 nm. For samples with t_N_ ≥ 1.2 nm, the FMR spectra are sharp with fairly narrow peak-to-peak linewidth (W_pp_ < 10 mT) that permits resolving small linewidth changes of ~0.1 mT.

**Figure 2:**
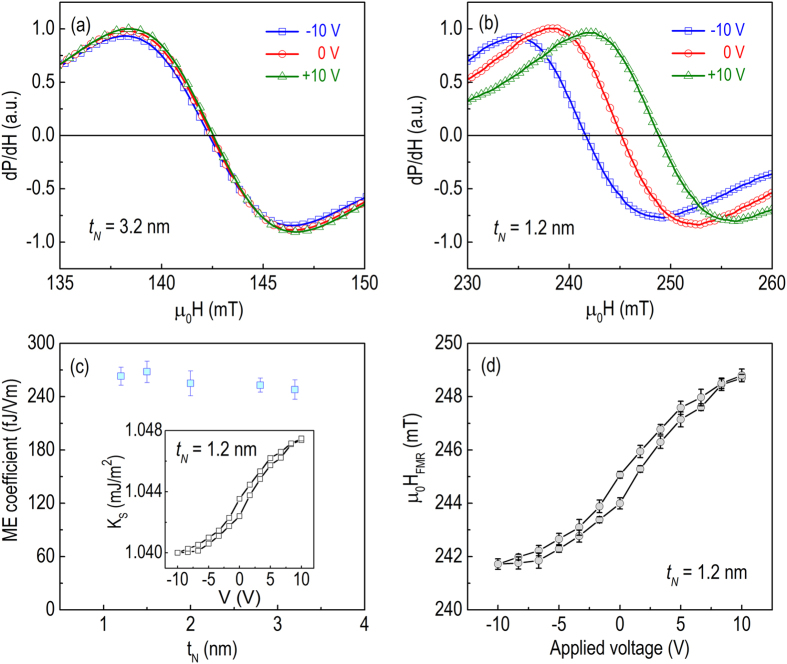
Modification of FMR spectra by constant voltages. (**a**,**b**) Voltage-induced shift of FMR spectra for NiFe/PLZT with (**a**) t_N_ = 3.2 nm and (**b**) t_N_ = 1.2 nm. (**c**) Dependence of the ME coupling coefficient on NiFe thickness t_N_, the inset is the magnetic surface anisotropy K_S_ as a function of applied voltage. (**d**) Resonance field as a function of applied voltage for NiFe/PLZT with t_N_ = 1.2 nm.

Modification of FMR spectra by constant dc voltages of opposite polarity is shown in Fig. 2. The shift of resonance field (μ_0_ΔH_FMR_) at applied voltages of ±10 V is small for NiFe/PLZT with t_N_ = 3.2 nm (Fig. 2(a)). When t_N_ = 1.2 nm, μ_0_H_FMR_ increases from 241.7 mT at −10 V to 248.8 mT at +10 V, with a pronounced modification of resonance field μ_0_ΔH_FMR_ = 7.1 mT (Fig. 2(b)). The voltage-induced resonance field change decreases as the NiFe thickness t_N_ increases from 1.2 to 3.2 nm, which reveals that the interfacial effect plays an important role, which is consistent with recent phenomenological theory and experimental study on multiferroic bilayer structures^43^,^44^,^46^,^47^,^48^. Figure 2(d) shows the resonance field as a function of applied voltage for NiFe/PLZT with t_N_ = 1.2 nm. The sample displays hysteresis behaviour that follows the polarization-voltage curve of PLZT showing in Fig. 1(c).

## Discussion

As to the origin of the voltage-induced shift of resonance field, one possibility is that a strain effect causes the magnetic anisotropy change by the application of piezo-strain to the magnetic layer. The strain is generated by piezoelectric effect in the ferroelectric layer and transferred to magnetic layer through mechanical interaction, which results in magnetic property change due to magnetostrictive effect, as has been shown for FeGaB/PZNPT^19^, FeGaB/PIN-PMN-PT^37^, and NiFe/PMNPT^38^ heterostructures. The other possibility is that an interfacial charge effect causes the magnetic anisotropy change. More specifically, the influence of voltage on the resonance field arises from charge accumulation at the magnetic/ferroelectric interface, thereby inducing unequal screening for spin-up and spin-down electrons in the magnetic layer that changes the surface anisotropy for the few atomic layers near the interface^11^. The strength of the charge-mediated ME coupling is therefore proportional to the surface charge accumulation, or rather, the polarization of the ferroelectric layer^44^. Moreover, since the surface anisotropy of ultrathin magnetic film is dominant in the total magnetic anisotropy energy, the anisotropy change would be more significant in thinner magnetic films. In contract, the anisotropy change in strain-induced ME coupling is independent on magnetic film thickness.

**Figure 3:**
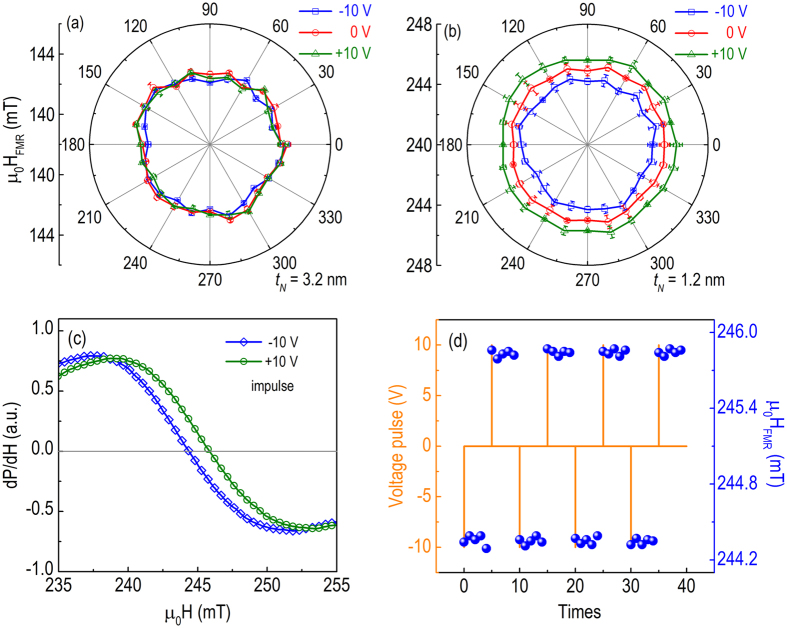
Non-volatile ferroelectric switching of ferromagnetic resonance by voltage pulses. (**a**,**b**) Angular dependence of resonance field at various voltage pulses for NiFe/PLZT with t_N_ = 3.2 nm (**a**) and t_N_ = 1.2 nm (**b**). (**c**) Voltage-pulse-induced shift of FMR spectra for NiFe/PLZT with t_N_ = 1.2 nm. (**d**) Reversible, non-volatile resonance field shift induced by voltage pulses for NiFe/PLZT with t_N_ = 1.2 nm.

In order to distinguish the origin of the ME coupling effect in NiFe/PLZT, angular dependence of in-plane FMR spectra was acquired at different voltages. If the strain effect dominates, the anisotropic piezo-strain distribution inherent in the (111)-oriented ferroelectric thin film would induce anisotropic ME coupling, implying that the resonance field modification would have different values along different in-plane directions^38^. In contract, if the charge effect dominates, due to the highly isotropic in-plane distribution of charge carriers, the resonance field shift measured in the film plane would be essentially equivalent^44^. Figure 3(a,b) show the angular dependence of μ_0_H_FMR_ for NiFe/PLZT with t_N_ = 3.2 and 1.2 nm, respectively. There is no clear shift in resonance field at any angle for the sample with t_N_ = 3.2 nm, meaning that the anisotropy change is negligible. When t_N_ reduces to 1.2 nm, an isotropic μ_0_ΔH_FMR_ of 1.6 mT is observed under the application of voltage pulses (±10 V). This μ_0_ΔH_FMR_ keeps constant along all in-plane angles, indicating that the strain effect should be negligible, and the resonance field shift might be induced by a perpendicular (out-of-plane) magnetic anisotropy change due to the interfacial charge effect^44^. This is understandable since, on one hand, both the piezoelectric coefficient (−e_31,f_ < 8 C/m^2^)^49^ of (111)-oriented PLZT film and the magnetostriction (λ_s_ < 1 ppm for t_N_ = 1.2 nm)^50^ of ultrathin NiFe are very small, permitting a convenient assumption of zero strain-mediated ME coupling when interfaced together; on the other hand, the resonance field modification described in Figs 2 and 3(a,b) is thickness dependent and strongly related to the polarization charges trapped at the NiFe/PLZT interface, consistent with an interfacial charge-mediated ME coupling. In contract, the anisotropy change of strain-induced ME coupling would be independent on magnetic film thickness within this thickness range. For example, we do not observe any clear shift in resonance field for the sample with t_N_ = 3.2 nm, meaning that the strain-mediated ME coupling is negligible. Note that the PLZT films are granular, so the anisotropy in the voltage-induced strain may be destroyed or reduced by the presence of differently oriented grains. Therefore, a strain effect may still be present within each grain but averaged out for the whole film. Based on the experimental evidence, we believe that the charge effect is the dominant mechanism for the ME coupling, while the strain effect is negligible in ultrathin NiFe/PLZT heterostructures.

The resonance field can be modified by applying voltage pulses (Fig. 3) instead of constant voltages (Fig. 2). The non-volatile modification of resonance field is further investigated for the sample with t_N_ = 1.2 nm, as shown in Fig. 3(c,d). By applying voltage pulses of opposite polarity with amplitude of 10 V and duration of 100 ms, a non-volatile and reversible resonance field switching of 1.6 mT is obtained, arising from the two remanent polarization states within the PLZT thin films. The ferroelectric PLZT thin film acts as a capacitor and is charging under the application of voltages. After removing the external voltage an internal electric field induced by the remanent polarization remains and continues to modify the magnetic anisotropy of the NiFe layer^12^,^44^. Therefore, the resonance field remains modified until the next pulse is applied due to long retention time of the ferroelectric layer, implying non-volatile functioning of NiFe/PLZT ME devices that stems from the ferroelectric layer. Note that the resonance field changes are different for the case of static voltage drive (Fig. 2(d)) and the case of pulse voltage drive (Fig. 3(d)), which is due to the two ferroelectric polarization states, i.e., saturation polarization (P_s_) and remanent polarization (P_r_) of PLZT thin film. When static voltage 10 V is applied, the ferroelectric capacitor is charging to saturation state. When voltage pulse 10 V is applied, the ferroelectric capacitor is rapidly charging to saturation state, and then reducing to remanent polarization state. Higher surface charge density is expected for static voltage since P_s_ (35 μC/cm^2^) is much larger than P_r_ (7 μC/cm^2^) in PLZT, which would induce a larger modification on the magnetic anisotropy of the NiFe layer due to charge-mediated ME coupling. In contract, voltage pulse would lead to lower ME coupling, but this process is non-volatile and consumes lower power, which are beneficial for device applications.

Having established that electrostatic screening is responsible for the observed ME effect and considering that the modifications of resonance field arises from the perpendicular magnetic anisotropy change, we can quantify the charge-induced surface anisotropy change ΔKs(V) using the energy equation^11^,


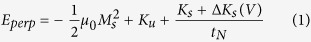


where E_perp_ is the perpendicular magnetic anisotropy energy, μ_0_ is the permeability of free space, μ_0_M_S_ = 1.1 T is the saturation magnetization of 1.2 nm NiFe^38^, K_u_ is the bulk anisotropy, K_S_ is the surface anisotropy, and t_N_ is the magnetic film thickness. Following the resonance condition and minimizing the total energy of magnetic films, the resonance field H_FMR_ is then determined by the Kittel equation,


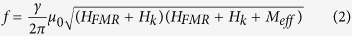


where f is the resonance frequency, γ/2π = 28 GHz/T is the gyromagnetic ratio, H_k_ = 2K_u_/μ_0_M_S_ is the bulk anisotropy field, and M_eff_ is the effective saturation magnetization incorporating out-of-plane magnetic anisotropy,


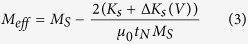


At a fixed frequency f = 9.76 GHz, the change in resonance field ΔH_FMR_ induced by the charge effect is solved as,


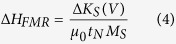


Clearly the change in resonance field is isotropic and dependent on the magnetic surface anisotropy change, consistent with the results shown in Fig. 3. The magnetic surface anisotropy K_S_ and the ME coupling coefficient is calculated and shown in Fig. 2(c). Under the application of ±10 V constant voltages, the change in perpendicular surface anisotropy is estimated to be 7.5 μJ/m^2^ for NiFe/PLZT films with t_N_ = 1.2 nm. This would correspond to a giant electric field effect on magnetic anisotropy of 263 f J/Vm, higher than the experimental results of 30–50 f J/Vm and theoretical prediction of 100 f J/Vm (10^−8^ erg/V cm) for Fe/MgO and FeCoB/MgO system^40^,^51^,^52^. However, for PLZT with a large dielectric constant of 1500, an even larger ME coupling coefficient is expected in NiFe/PLZT based on interfacial charge effect. Recently it has been reported that NiO clusters may form at the NiFe/PMN-PT interface due to oxygen diffusion, which was confirmed by EELS and EDS measurements^53^. Similarly, NiO could form at the NiFe/PLZT interface with a significantly lower dielectric constant, which might be the reason for the low experimental value. No significant NiFe thickness dependence of ME coupling coefficient is observed over the measured thickness range, which is consistent with previous reports^51^,^52^, indicating that the electric field control of magnetic anisotropy originates primarily from the NiFe/PLZT interface. Most remarkably, ΔK_S_ = 1.7 μJ/m^2^ is achieved under the application of ±10 V voltage pulses at room temperature, comparable to the previously reported value in the Fe/MgO heterostructures^11^. In the first demonstration in Fe/MgO, the charge effect was produced by applying relatively high voltages of ±200 V, and persisted only as long as the voltage was applied^11^. Later the voltage was reduced to less than 1 V by removing the thick polyimide layer^54^. The non-volatile ferroelectric switching of magnetic anisotropy in NiFe/PLZT allows short voltage pulses instead of constant voltages, to manipulate magnetic parameters in multiferroic heterostructures, which would significantly enhance the power efficiency for integrated RF and spintronic devices.

## Conclusions

In summary, our work shows that magnetic anisotropy of ultrathin magnetic films can be electrically modified in magnetic/ferroelectric bilayers at low voltage. Specifically for NiFe/PLZT bilayers, where perpendicular magnetic anisotropy is sensitive to interfacial charge accumulation and depletion, we use voltage control of interfacial charge screening to achieve control over magnetic anisotropy. In fact, this piezoelectric-free structure provides better reliability and longer lifetime for device applications since piezoelectric deformation potentially leads to fatigue and fracture over time. Moreover, we show that by simply applying voltage pulses, a robust, non-volatile, and reversible modification of magnetic anisotropy is demonstrated due to the remanent polarization in the ferroelectric layer. Therefore, considerable further improvement in non-volatile performance and functionality can probably be anticipated by examining ferroelectrics with higher remanent polarization such as PZT, BiFeO_3_, or doped HfO_2_. Our results thus provide a pathway towards ferroelectric switching of magnetism that could be useful for compact, reconfigurable, and energy-efficient tunable RF and spintronic devices.

## Methods

### Sample preparation

Ferroelectric PLZT thin films were prepared by chemical solution deposition on platinized silicon Pt/Ti/SiO_2_/Si(001) wafers. After spin-coating, the PLZT films were pyrolyzed at 450 °C for 10 min and crystallized at 650 °C for 15 min. The film thickness was estimated to be 350 nm. To check the electrical properties, Pt top electrodes with a diameter of 250 μm and a thickness of 100 nm were deposited on PLZT films by electron-beam evaporation. For magnetic layer deposition, the PLZT thin films were loaded into a magnetron sputtering chamber with a background pressure of ~1 × 10^−7^ Torr. Plasma etching was carried out to clean the surface and reduce the roughness. NiFe dots, with a diameter of 500 μm and nominal thicknesses t_N_ = 1.2, 1.5, 2.0, 2.8, and 3.2 nm, were DC sputtered onto the PLZT films through a shadow mask at room temperature under 3 mTorr Ar. 5 nm Cu was then deposited on top at the same condition as the capping layer. The uncertainty in the film thickness was estimated to be <10% from x-ray reflectivity.

### Structural, electrical, and ferromagnetic characterization

Phase identification was performed on a Bruker D8 AXS x-ray diffractometer with Cu Kα radiation. Microstructure and surface morphology of PLZT were examined by Atomic Force Microscope (AFM). Dielectric measurements were conducted with an Agilent E4980A LCR meter using an oscillator level of 0.1 V in conjunction with a Signatone QuieTemp probe station. Polarization vs applied voltage (P-V) loops of PLZT thin films were measured at a maximum applied voltage of 10 V and frequency of 100 Hz on a Radiant Technologies’ Precision Premier II tester. Ferromagnetic resonance (FMR) spectra were measured using a Bruker EMX electron paramagnetic resonance (EPR) spectrometer with a TE_102_ cavity, operated at a microwave field frequency of 9.76 GHz and power of 10 mW. The voltage-dependent FMR spectra were obtained by applying voltages across the film thickness direction during FMR field sweeping. The 100 ms voltage pulses used in the experiments were controlled by an electric relay. For angular-dependent FMR spectra measurements, the NiFe/PLZT bilayers were attached to a sample holder with a precise angle rotator. All measurements were conducted at room temperature.

## Additional Information

**How to cite this article**: Hu, Z. *et al*. Non-Volatile Ferroelectric Switching of Ferromagnetic Resonance in NiFe/PLZT Multiferroic Thin Film Heterostructures. *Sci. Rep.* **6**, 32408; doi: 10.1038/srep32408 (2016).
